# Additional Prognostic Value of Tissue Doppler Evaluation in Patients with Aortic Stenosis and Left-Ventricular Systolic Dysfunction Undergoing Aortic Valve Replacement

**DOI:** 10.3390/medicina58101410

**Published:** 2022-10-07

**Authors:** Luminita Iliuta, Andreea Gabriella Andronesi, Camelia Cristina Diaconu, Eugenia Panaitescu, Georgiana Camburu

**Affiliations:** 1 Department of Medical Informatics and Biostatistics, University of Medicine and Pharmacy “Carol Davila”, 050474 Bucharest, Romania; 2 Cardioclass Clinic for Cardiovascular Disease, 031125 Bucharest, Romania; 3 Nephrology Department, University of Medicine and Pharmacy “Carol Davila”, 050474 Bucharest, Romania; 4 Nephrology Department, Fundeni Clinical Institute, 022328 Bucharest, Romania; 5 Internal Medicine Department, University of Medicine and Pharmacy “Carol Davila”, 050474 Bucharest, Romania; 6 Internal Medicine Clinic, Clinical Emergency Hospital of Bucharest, 014461 Bucharest, Romania; 7 Academy of Romanian Scientists, 3 Ilfov Street, 050044 Bucharest, Romania

**Keywords:** aortic stenosis, systolic dysfunction, aortic valve replacement, restrictive diastolic filling pattern

## Abstract

*Background and Objectives*: Patients with surgical aortic stenosis (AS) show impaired diastolic filling, which is a risk factor for early and late mortality after aortic valve replacement (AVR). There is a paucity of information concerning the impact of restrictive diastolic filling and the evolution of diastolic dysfunction in the early and medium terms post-AVR. We aimed to determine the prognostic value of the presence of a restrictive left-ventricular (LV) diastolic filling pattern (LVDFP) and dilated left atrium (LA) in patients with AS and LV systolic dysfunction (LVEF < 40%) who underwent AVR, and to define the independent predictors for immediate and long-term prognosis and their value for preoperative risk estimation. *Materials and Methods*: The study was prospective and included 197 patients with surgical AS and LVEF <40% who underwent AVR. Preoperative echocardiographic examinations were repeated at day 10, at 1, 3 and 6 months, and at 1 and 2 years after surgery, with evaluation of LVEF, diastolic function and LA dimension index (mm/m^2^). Depending on LV systolic performance, patients were classified as Group A (LVEF: 30–40%) or Group B (LVEF < 30%). *Results*: The main echographic independent parameters for early and late postoperative death were: restrictive LVDFP, significant pulmonary hypertension, LV end-systolic diameter (LVESD) >55 mm and the presence of second-degree mitral regurgitation. Restrictive LVDFP and LA dimension >30 mm/m^2^ were independent predictors for fatal outcome (*p* = 0.0017). *Conclusions*: Assessment of diastolic function and LA dimension are reliable parameters in predicting fatal outcome and hospitalization for heart failure, having an independent and incremental prognostic value in patients with surgical AS. Complete evaluation of LVDFP with all the echographic measurements (including TDI) should routinely be part of the preoperative assessment of patients with LV systolic dysfunction undergoing AVR.

## 1. Introduction

The most frequent cardiovascular diseases that affect the elderly population are: hypertension, coronary artery disease and aortic stenosis (AS), which is also the most common valvular heart disease [1]. Its prevalence has increased in recent years (from 2% in adults over 65 years to 4% in adults over 85 years of age) [2], with increasing life expectancies and standards of living in Western countries having a great impact on mortality and morbidity in the elderly population [1]. 

The risk of myocardial-infarction- and cardiovascular-caused death is increased by 50 percent if AS is associated, despite the non-hemodynamically significant obstruction of the LV outflow tract [3,4].

As long as the increased wall thickness can compensate the high systolic pressure from the inside of the LV, the systolic function of the LV in patients with AS will be preserved [5]. If the compensatory mechanisms are not effective (depressed LV contractility or inadequate hypertrophic processes), LV systolic dysfunction gradually develops [6]. There are studies which have demonstrated that the systolic performance of LV has an important influence on the postoperative evolution of patients with AS undergoing AVR, and the presence of severe systolic dysfunction is associated in general with a more unfavorable postoperative evolution [7]. On the other hand, diastolic dysfunction has been demonstrated to precede the alteration in systolic function in patients with LV hypertrophy caused by aortic valve disease [8,9]. 

Although many studies on diastolic dysfunction have been published, there is a paucity of information on diastolic dysfunction evolution in the early and medium terms after aortic valve replacement. Only a few studies have evaluated the real impact of the type of LV diastolic filling pattern (LVDFP) on early postoperative evolution in patients with surgical AS [10,11]. Previous research has revealed that the presence of a restrictive LVDFP determines a more unfavorable prognosis in all cardiac diseases. There are only a few studies that have evaluated the influence of restrictive LVDFP and LA dilatation on early postoperative evolution in patients with AS undergoing AVR [12]. These studies are largely heterogeneous, with relatively small populations and variable patient selections; they have evaluated outcomes, type of surgical procedure, and length of postoperative follow-up, thus limiting the applicability of individual reports [13,14,15]. However, there have been no serial long-term studies of the effect of AVR on LV diastolic function, and the relationship between the reversal of LV dilatation magnitude and the increase in LV diastolic performance has not been studied intensively.

To address these issues, we studied a series of patients undergoing AVR for chronic AS. Severity of disease was estimated by symptoms, clinical evaluation, echocardiography and cardiac catheterization. The main objective of our study was to assess the immediate prognostic implications of the LVDFP in patients with AS undergoing AVR. We also identified the echographic parameters which can be considered independent predictors for short- and long-term outcomes in these patients and their adjusted values for the evaluation of preoperative risk.

## 2. Patients and Methods

### 2.1. Study Population

We performed a prospective study of 197 adult patients (mean age: 70 ± 12 years) with surgical AS, diagnosed by two-dimensional and Doppler transthoracic echocardiogram and LVEF <40% (mean LVEF: 28 ± 5%), who underwent AVR. Patients were evaluated clinically and by heart ultrasound before the surgical intervention and at 10 days and 1, 3, 6, 12 and 24 months postoperatively. For each patient, systolic and diastolic LV performance were evaluated, along with LA function, the severity parameters of AS and the dimensions of the heart chambers. 

Written informed consent was obtained from all patients. Patients were asked by letter to agree to follow-up visits on an ambulatory basis. The study protocol was approved by the local ethics committee.

### 2.2. Objectives

1. To assess the independent prognostic value of the presence of a restrictive mitral flow pattern and dilated LA in patients with surgical AS and altered LV systolic function undergoing AVR.
2. To determine short- and long-term-prognosis echographic independent predictors, with their adjusted values used for preoperative risk evaluation.

We analyzed the following endpoints:

- Type of LVDFP;
- Quality of life (measured on a scale from 1 to 10 using a questionnaire that was filled in at each visit by the patient);
- Death.

We evaluated global quality of life, including a mental component (MCS) and a physical component (PCS), using a self-report questionnaire. The scores were continuous from 0 (lower) to 10 (higher quality of life). Patients outlined their change in quality of life at the 30-day follow-up questionnaire by answering from both points of view, mental and physical, the question: “How would you rate your quality of life now?”. The answer choices were: “Worse than before your procedure”, “The same as before your procedure” and “Better than before your procedure”.

Exclusion criteria:

- Associated or previous mitral or tricuspid valve replacement or repair;
- Aortic dissection;
- Previous aortic valve surgery;
- Congenital diseases unrelated to AS;
- Coronary significant lesions (more than 50% reduction in luminal diameter);
- Postoperative prosthesis mismatch;
- Permanent atrial fibrillation;
- Presence of a pacemaker;
- Left or right bundle-branch block.

Patients undergoing concomitant procedures, such as associated ascending aortic surgery with AVR, were not excluded.

Depending on LV systolic performance, the patients were divided into two groups:

- Group A—136 pts, with moderate systolic dysfunction and LVEF = 30–40%;
- Group B—61 pts, with severe systolic dysfunction and LVEF <30%.

### 2.3. Ultrasound Methods

The diagnosis of AS was performed using primary hemodynamic parameters recommended for evaluation: AS jet velocity, mean transaortic gradient and the valve area by continuity equation [16] (Table 1).

All echocardiographic examinations were performed with a Philips Affinity 30 or a portable General Electric VIVID machine, with a 3.5 MHz probe. Echocardiographic techniques and calculations were performed in accordance with the recommendations of the European and American Society of Echocardiography [17].

The parameters assessed at each visit were: LV systolic and diastolic performance (including TDI measurements), LA indexed volume, aortic prosthesis hemodynamic parameters and dimensions of the heart cavities (LVEDV, LVESV, LA) [18].

Left-ventricular ejection fraction (LVEF) was calculated using a modified Simpson’s method. The assessment of diastolic function was based on a comprehensive echocardiographic study integrating all available two-dimensional and Doppler data. By placing the pulsed-wave Doppler between the mitral leaflet tips in the apical four-chamber view, we could record the transmitral flow. We measured the transmitral peak early-diastolic (E) and late (A)-flow velocities, the E/A ratio and the deceleration time (EDt) [18]. To calculate the isovolumetric relaxation time (IVRT), we placed the pulsed-wave Doppler cursor in the area of the anterior mitral valve leaflet to capture an LVOT envelope and the mitral inflow profile. The interval from the aortic-valve artifact at the end of the LVOT envelope to the mitral-valve artifact at the beginning of the mitral E wave was considered to be the IVRT. In addition, using pulsed-wave Doppler, we measured pulmonary venous flow: “forward” systolic (S) and diastolic (D) velocities into the left atrium, and the “backward” late-diastolic A reversal wave corresponding to atrial contraction.

We also performed tissue Doppler imaging (TDI) by recording myocardial longitudinal velocities at the mitral annulus, with the sample volume (2–5 mm) placed at the lateral border of the mitral annulus in the apical four-chamber view for estimation of LV filling pressures. The lateral mitral annulus was aligned parallel to the sampling cursor, and the sweep speed was set at 50 mm/s. The spectral Doppler-signal filter settings were adjusted at the lowest wall filter and at the minimum optional gain. We measured:

- Peak annular systolic velocity (Sa) as the peak negative systolic wall motion wave at the level of the T-wave of the ECG;
- Early-diastolic velocity (Ea), which reflects the velocity of early myocardial relaxation as the mitral annulus ascends during early rapid LV filling;
- Late-diastolic (Aa) velocity.

**Figure 1:**
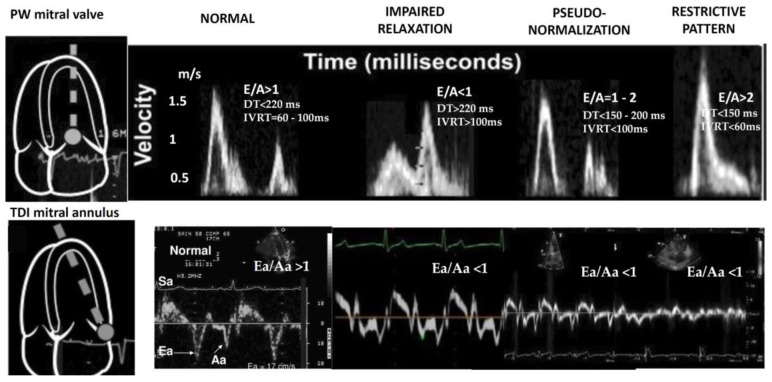
Diastolic filling patterns evaluated by pulsed-wave Doppler and TDI.

The classification of the diastolic function was: normal (E/A > 1, DT < 220 ms, IVRT = 60–100 ms and Ea/Aa > 1), impaired relaxation (E/A < 1, DT > 220 ms, IVRT > 100 ms and Ea/Aa < 1), pseudonormalization (E/A = 1–2, DT = 150–200 ms, IVRT < 100 ms and Ea/Aa < 1) and restrictive pattern (E/A > 2 or DT < 150 ms, IVRT < 60 ms and Ea/Aa < 1) (Figure 1).

All patients over 35 years of age, as well as patients under 35 years old with angina pectoris, underwent arteriography. None of the patients had associated coronary artery disease (>50% reduction in luminal diameter of any coronary artery). For patients presenting with symptoms suggestive of heart failure and preserved systolic function, brain natriuretic peptide (BNP) titration was performed. Diastolic heart failure was considered unlikely for values under 35 pg/mL and possible if values surpassed 100 pg/mL.

### 2.4. Statistical Analysis

Statistical analyses were performed using SYSTAT and the Statistical Package for the Social Sciences 11.5 (SPSS 11.5) software. Continuous variables were indicated as means ± standard deviations, and qualitative data were recorded as percentages. Categorical variables were tested using the Pearson chi-square test, likelihood ratios and Fisher’s exact test, and quantitative data between the two groups were tested with the independent-samples *t*-test, while data between three groups were tested with analysis of variance (ANOVA) and Tukey’s post hoc test, assuming equal variances, and the Games–Howell post hoc test, assuming nonequal variances between the groups. The Wilcoxon test and the Friedman test were used to compare preoperative and postoperative echocardiographic variables. 

The association between preoperative variables and postoperative change in LVEF was based on Pearson correlation analysis, and linear regression analysis was used to establish the associations between preoperative data and the parameters associated with postoperative evolution (death, NYHA class and quality of life). Univariate logistic regression analysis was used to compare the two groups. Multivariate logistic regression analysis was performed to identify predictive factors of mortality. Areas under the receiver-operating-characteristic (ROC) curves and Hosmer–Lemeshow goodness-of-fit statistics were calculated to assess the discrimination and calibration of the model, respectively. To evaluate the goodness-of-fit of the model, Cox–Snell/Nagelkerke values were calculated. A probability value of <0.05 was considered statistically significant.

We tested two main hypotheses. The first was that LA dilatation and restrictive LVDFP are independent predictors for unfavorable postoperative evolution in patients with AS and LV systolic dysfunction undergoing AVR. The second hypothesis tested was that the presence of restrictive LVDFP makes a higher contribution than LV systolic dysfunction to preoperative risk calculations for patients with AS undergoing AVR. For hypothesis testing, we used univariate and multivariate logistic regression analysis, correlation-coefficient calculations and nonparametric Friedman testing (which indicated the differences between the three analyzed moments—preoperative, early postoperative and 2 years postoperative).

## 3. Results

Taking into account the postoperative course of the study groups, we calculated the overall percentage of patients whose self-reported quality-of-life scores (SR QOL scores), changed between better/the same/worse (including stratification by self-reported global, mental and physical scores). In group A, 48.53% of patients had a preoperative self-reported quality-of-life score less than 5 compared to 85.24% of patients from group B (*p* < 0.005, likelihood ratio). In addition, at 30 days postoperatively, 60,29% of patients from group A reported a better postoperative quality-of-life score compared with only 36.06% in group B with preoperative severe LV systolic dysfunction (*p* < 0.005, likelihood ratio). 

Postoperative echocardiography showed a trend toward improvement in LVEF in group B (*p* = 0.06) and significant improvement in group A (*p* = 0.002). Postoperative end-diastolic and end-systolic dimensions decreased significantly in both groups (Table 2).

**Figure 2:**
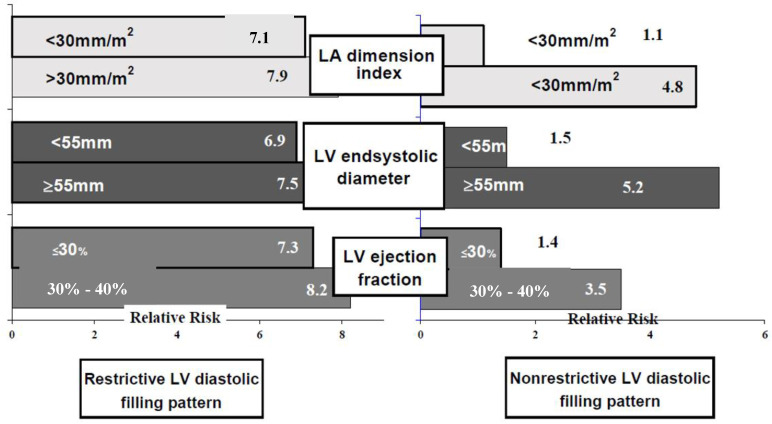
The early risk of death depending on the LVDFP in patients with AS and LV systolic dysfunction undergoing AVR.

Simple and multivariate regression analysis showed that the presence of restrictive LVDFP (EDt < 150 ms, E/A > 2) increased the risk of death early after surgery, regardless of the presence of other parameters known to increase mortality rates in patients with AS undergoing surgical treatment. The analysis of the two groups, depending on the type of LVDFP, revealed that the restrictive LVDFP turned out to be an independent predictor for increased early mortality rates in these patients (*p* = 0.001), regardless of LV dimensions and systolic function, patient age, comorbidities and presence of pulmonary hypertension (Figure 2). There were 40 patients in group A and 22 patients in group B with restrictive LVDFP. Multivariate logistic regression analysis showed that, in patients with non-restrictive LVDFP, the prognostic factors are: LA dimension index, LV end-systolic diameter and LVEF. For patients with the restrictive pattern, regardless of the other factors involved, the prognosis is worst.

In addition, the index of LA dimension >30 mm/m^2^ was also found by simple and multivariate regression analysis to be an independent predictor for fatal outcome in patients with AS and LV systolic dysfunction undergoing AVR (RR = 7.2, *p* = 0.0017), but mostly in those with restrictive LVDFP. The prognostic value of LA dilatation decreases if there is a concomitantly pulmonary hypertension or a second-degree mitral regurgitation. In these patients, the presence of LA dilatation was not correlated with a higher early postoperative mortality rate (r = 0.21, *p* < 0.05). 

The presence of a restrictive LVDFP homogenized the relative risk. In patients with this type of filling pattern, early postoperative risk of death was increased regardless of LV systolic performance, patient age or the presence of LV hypertrophy with an interventricular septum thickness between 12 and 18 mm. 

Severity of AS and LV diastolic dimensions were not correlated with increased early postoperative mortality.

Independent echographic predictors for early postoperative mortality were determined by simple and multivariate regression analysis:

- The presence of second-degree MR (RR = 12.6, r = 0.62, *p* < 0.05);
- Isovolumetric relaxation time <60 ms (RR = 10.9, r = 0.71, *p* < 0.05);
- E-wave deceleration time <100 ms (RR = 10.8, r = 0.75, *p* < 0.05);
- S/D ratio <1 in pulmonary venous flow (RR = 10.9, r = 0.55, *p* = NS);
- Mean pulmonary artery pressure >50 mmHg (RR = 9.7, r = 0.74, *p* < 0.05);
- LV end-systolic diameter (LVESD) >55 mm (RR = 8,6, r = 0.65, *p* = 0.062).

Parameters of LV systolic function, end-systolic and end-diastolic diameters and volumes, as well as parameters of aortic lesion severity (gradient between LV and aorta, aortic annulus area) were not correlated with increased early postoperative mortality in these patients (r = 0.2, *p* = 0.052).

The cut-off value for E-wave deceleration time <100 ms was estimated using both the classical method of two standard deviations from the difference between the mean values for restrictive and non-restrictive LVDFP and ROC curves, taking into account the maximization of sensitivity. For an E-wave deceleration time <150 ms, the associated relative risk was only 5.6, and it was not significantly correlated with postoperative mortality (r = 0.46, *p* = NS).

**Figure 3:**
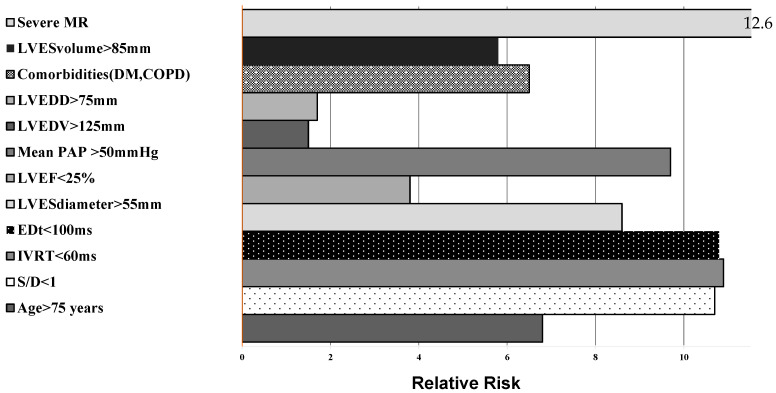
Early risk of death in patients with AS and LV systolic dysfunction undergoing AVR.

The relative risks associated with early postoperative death and the parameters which increase mortality rates in AS are presented in Figure 3. Risk of early postoperative death was augmented about 10-fold by the presence of the restrictive diastolic filling pattern (EDt <100 ms by 10.8-fold and IVRT <60 ms by 10.9-fold), 10.7 fold by a ratio of systolic to diastolic waves in the pulmonary veins less than 1, 4.2-fold by eccentric LV hypertrophy with interventricular septum thickness >18 mm, and 6.8-fold by patient age >75 years. In addition, the presence of pulmonary hypertension, associated moderate mitral regurgitation and associated comorbidities (diabetes mellitus, chronic obstructive pulmonary disease) increased the relative early postoperative risk values and were associated with higher mortality rates (r between 0.62–0.71, *p* < 0.05).

The factors associated with unfavorable evolution in patients with AVR at 2 years after surgery revealed by multivariate logistic regression analysis were: age >75 years (RR = 6.3, *p* < 0.01), end-diastolic volume >85 cm^3^ (RR = 5.2, *p* < 0.0001), restrictive LVDFP (RR = 7.9, *p* < 0.002) and LV eccentric hypertrophy (RR = 6,9, *p* = 0.005). Patients with LV concentric hypertrophy had a better prognosis than patients with eccentric hypertrophy (RR = −2.7, *p* = 0.001).

Regarding self-reported quality of life at 2 years postoperatively, the percentage of patients with a better postoperative quality-of-life score in group A was also significantly higher compared with group B (67.5% vs. 31.7%, *p* < 0.005, likelihood ratio). Taking into account the presence of a restrictive LVDFP, Friedman testing showed that it has a significantly higher influence on quality-of-life score at 2 years postoperatively compared with LV systolic severe dysfunction (LVEF <30%). The percentage of patients with a better postoperative quality-of-life score in the nonrestrictive LVDFP group was 72.22% compared with only 17.07% in the group with preoperatively restrictive LVDFP (*p* < 0.005, likelihood ratio).

The predictive value for death at 2 years postoperatively of LV systolic dysfunction, age >75 years and interventricular septum thickness were higher in the nonrestrictive LVDFP group of patients. In these patients, LVEF <35%, age >75 years and interventricular septum thickness >18 mm increased the risk of death at 2 years postoperatively.

## 4. Discussion

Diastolic dysfunction is one among a variety of comorbid conditions found in a significant number of patients who undergo cardiac surgery. Their postoperative courses can be complicated by abnormalities in diastolic function leading to diastolic heart failure and which occur more commonly in the elderly and in patients with diabetes mellitus and hypertensive or valvular heart disease, as well as in patients with hypertrophic and restrictive cardiomyopathy [19,20]. Despite being a common cause of heart failure, it still remains under-reported postoperatively [21]. 

During AVR, abnormal diastolic filling patterns are frequently noticed and more often in patients with altered LV function. The LV relaxation constant, LV systolic function, LV end-diastolic volume, intrinsic myocardial stiffness and left atrial pressure are several determinants that influence impaired diastolic filling in patients with clinically significant AS [21], which is a risk factor for early and late mortality after surgery [22]. 

An increased risk of postoperative complications and vasoactive support in postoperative ICU settings may be associated with diastolic dysfunction, which presents an a priori risk for cardiopulmonary bypass [23,24].

The aim of the study was to estimate a series of long-term changes in LV thickness, dilatation and systolic and diastolic function and to evaluate the preoperative and early postoperative determinants of subsequent late-postoperative NYHA class, ventricular function mortality rate and quality of life. 

Nevertheless, the clinical question of whether it is too late to operate when diastolic function is severely depressed still remains open. The authors of the most recent reports in the literature for this field could not indicate when to recommend AVR for patients with a restrictive LVDFP [25]. There is still no clear definition of thresholds in the determination of a marked reduction in LV diastolic function in patients with surgical AS. Furthermore, the outcomes of patients with restrictive diastolic filling are not very well known because of leakage in a large number of patients with severe LV diastolic dysfunction, according to most studies [26,27]. 

LV afterload, myocardial contractility, the extent of irreversible interstitial fibrosis and chamber architecture are a number of preoperative factors on the complex interaction of which LV function depends after AVR [28]. Normalization of LV diastolic dysfunction has been reported in patients with severe AS after successful AVR [29,30].

Satpathy et al. reported that LV diastolic and systolic function and the regression of LV structure continue for decades after AVR, even though an immediate hemodynamic improvement is noticed after AVR [31].

It was reported that patients with an LA diameter of less than 3.55 cm had increased long-term survival compared to patients with a severely enlarged LA (≥5.0 cm in diameter) at 5 (85% vs. 61%) and 10 (62% vs. 28%) years after AVR (*p* = 0.006) [9]. Preoperative severe LV hypertrophy, together with left atrial dilatation (suggesting diastolic dysfunction), significantly reduced long-term survival, independently of symptom status [32]. Left-atrial dilatation with preoperative severe LV hypertrophy significantly decreases long-term survival, independently of symptom status [32]. 

In our study, the indices of LA dimension >30 mm/m^2^ for group A and >32 mm/m^2^ for group B were found to be independent predictors for fatal outcome (RR = 6.3, respectively RR = 8.2, *p* = 0.0017).

A study directed by Milano et al. emphasized that increased LV diameter and reduced LV function in patients with severe AS were associated with percentage of myocardial fibrosis, influencing long-term survival after AVR. These patients were found to have an increased risk of cardiac death at 10 years postoperatively as a result of congestive heart failure [28].

Moderate-to-severe diastolic dysfunction is still seen after 10 years post-AVR, despite reduction in LV mass index, according to Gjertsson et al. [23].

There is a relative increase in LV fibrous content soon after AVR and a relative reduction after 4 to 5 years, these changes occurring as part of a two-step process, with the regression of LV interstitial fibrosis leading to the reversal of LV diastolic function [23,29].

We noticed in our study that LV end-systolic diameter (LVESD) >55 mm was an echographic predictor of early postoperative mortality in patients with surgical AS and LV systolic dysfunction.

The exposure of the heart to ischemia and reperfusion after AVR may result in the worsening of systolic or diastolic dysfunction [32], requiring positive inotropic drugs and mechanical circulatory support, usually transient, but it may lead to post-operative complications and death [33]. Deterioration of LV compliance and relaxation are seen in more than 90% of patients with AS and precede systolic dysfunction in these patients [34,35]. In patients undergoing successful AVR, LV fibrosis progressively diminishes, but in the short term there is an increase in interstitial fibrosis compared with a decrease in hypertrophied myocytes [31]. Together with the myocardial stunning which accompanies every cardiac surgery, this increases the risk of short-term complications due to diastolic dysfunction after AVR. A reduced relaxation time due to increased LV fibrosis may lead to acute diastolic dysfunction after successful AVR, which is usually very difficult to manage [12]. In our study, a reduced E-wave deceleration time of less than 100 ms and a reduced isovolumic relaxation time of less than 60 ms—as markers of LV diastolic dysfunction—were independent predictors for early post-AVR mortality. Reversible restrictive filling may be converted to irreversible restrictive filling after AVR, especially in patients with severe preoperative diastolic dysfunction [36]. 

Elevated end-diastolic pressure is common in surgical AS and is related to pulmonary artery hypertension. Early and late outcomes after valve replacement are affected by the presence of pulmonary hypertension, which is considered a significant risk factor [37]. Operative mortality after AVR was significantly increased in patients with pulmonary hypertension (9% vs. 5%), which is an independent risk factor for decreased long-term survival [38].

The prognoses of patients with AS and severe pulmonary hypertension are very poor among patients treated conservatively [39], and AVR improves prognosis, although outcomes are worse in those with associated severe pulmonary hypertension [40]. In our study, pulmonary hypertension higher than 50 mmHg was an independent predictor for early postoperative mortality; these data suggest that pulmonary hypertension has a significant impact on outcomes in patients undergoing AVR and should be considered in preoperative assessment.

On the other hand, management of mitral regurgitation (MR) during AVR for AS of intermediate degree is still controversial. The associated MR in patients undergoing AVR is frequently not corrected because it may improve after AVR. However, it was proved that the reduction in MR after relief of AS is modest and that gradient change across the aortic valve has very little impact on the reduction in MR [41]. The impact of mitral regurgitation in patients undergoing AVR is not clearly defined. In a study by Barreiro et al., moderate MR was an independent risk factor for reduced long-term survival in elderly patients undergoing AVR [42]. In our study, grade-2 or worse MR was an independent risk factor for early post-AVR mortality. Therefore, we consider that patients with intrinsic mitral valve disease should be considered for concomitant MV surgery. It was noted in our study that if there is concomitant pulmonary hypertension or second-degree mitral regurgitation, the prognostic significance of LA dilatation decreases. 

Regarding quality of life, we observed that, although the patients with restrictive LVDFP survived, they performed much worse compared to the patients with the non-restrictive pattern, the presence of a restrictive LVDFP having a greater influence on the postoperative course compared with LV severe dysfunction.

However, defining the thresholds for a marked reduction in LV diastolic function in patients with surgical AS in order to calculate a preoperative risk score remains an unresolved issue. Our study provides evidence that severe diastolic dysfunction, in addition to LA dilatation, identifies patients at risk of poor prognosis after valvular aortic surgery. 

Clinicians should identify high-risk cardiac patients with the use of perioperative echocardiograms in ICUs and improve fluid and inotropic/lusitropic drug treatments postoperatively to provide proper treatment.

Preoperative diastolic dysfunction related to adverse cardiac outcome brings into question whether trials of specific perioperative strategies to enhance LV relaxation and filling patterns should be taken into account in patients undergoing AVR.

### Study Limits

In spite of this being one of the largest studies on tissue Doppler evaluation of diastolic filling in surgical AS, the patient number was still average.

Additionally, AVR, for all the patients in our study, was surgical and we did not have a comparison group of patients who underwent transcatheter aortic-valve implantation.

In summary, this study emphasizes that, for patients undergoing AVR, a full evaluation of diastolic function should routinely be part of echocardiographic assessment. 

## 5. Conclusions

The presence of a restrictive LVDFP can predict fatal outcome and heart-failure hospitalization in patients with surgical AS and LV systolic dysfunction undergoing AVR.

In these patients, LA dilatation had an incremental and independent prognostic value for early postoperative course assessment. In addition, a more reliable parameter for prognosis appreciation is LV diastolic performance compared with LV systolic function.

The presence of associated severe pulmonary hypertension or second-degree mitral regurgitation decreases the prognostic value of LA dilatation and increases mortality rates.

The presence of a restrictive LVDFP and severe pulmonary hypertension, second-degree mitral regurgitation and dilated LV with LVESD >55 mm can echographically anticipate augmented early postoperative mortality rates in patients with surgical AS and LV systolic dysfunction.

At 2 years postoperatively, age >75 years, LVESV >85 cm^3^ and the restrictive LVDFP were the only predictors for unfavorable evolution. Quality of life early and at 2 years post-AVR was significantly influenced by the presence of a restrictive LVDFP, which was more important than severe LV systolic dysfunction in prognostic prediction. 

Patient stratification has to take into account diastolic function in the first place, then heart-chamber dimensions, pulmonary hypertension and LV systolic performance. In order to calculate the preoperative risk score, it is very important to introduce guidelines regarding the presence of a restrictive pattern, which is as important as age and comorbidities in establishing prognoses. Present guidelines do not include pulse-wave and TDI diagnosis of the restrictive pattern in preoperative risk evaluation, and future updates could be necessary.

## Figures and Tables

**Table 1:** Baseline patient characteristics.

	Group A—136 pts LVEF = 30–40%	Group B—61 pts LVEF < 30%	*p*-Value
Mean (SD) age (years)	68 (12)	71 (11)	0.38 ^1^
Women	48 (35.29%)	22 (36.06%)	0.58 ^2^
Diabetes mellitus	80 (5.88%)	40 (6.56%)	0.35 ^2^
Current smoker	70 (5.15%)	30 (4.92%)	0.59 ^2^
Mean (SD) LVEF (%)	35 (5)	26 (4)	0.03 ^1^
Restrictive LV diastolic filling pattern	40 (29.41%)	22 (36.06%)	0.01 ^2^
Mean (SD) heart rate	75 (17)	74 (17)	0.64 ^1^
NYHA class I/II	40 (29.41%)	3 (4.92%)	0.001 ^3^
NYHA class III	72 (52.94%)	27 (44.26%)	
NYHA class IV	24 (17.65%)	31 (50.82%)	

LVEF—left-ventricular ejection fraction; LV—left ventricle; NYHA—New York Heart Association. ^1^ ANOVA. ^2^ Pearson chi-square. ^3^ Likelihood ratio.

**Table 2:** Comparison of preoperative and postoperative echocardiographic variables.

Echographic Variables	Group A—136 pts LVEF = 30–40%				Group B—61 pts LVEF < 30%			
	Before Surgery	6 Months after Surgery	2 Years after Surgery	*p* ^1^	Before Surgery	6 Months after Surgery	2 Years after Surgery	*p* ^1^
LV end-diastolic dimension (mm)	58 ± 6	54 ± 9	52 ± 8	0.08	62 ± 6	58 ± 8	57 ± 7	0.012
LV lateral-wall thickness (mm)	14 ± 0.2	13 ± 0.2	12 ± 0.2	0.04	17 ± 0.2	15 ± 0.4	13 ± 0.2	<0.001
IVS thickness (mm)	16.0 ± 0.4	15.4 ± 0.5	12.7 ± 0.7	0.05	18.0 ± 0.4	16.0 ± 0.2	14.4 ± 0.4	<0.001
Mean (SD) LVEF (%)	35 (5)	39 (12)	47 (10)	0.002	26 (4)	28 (5)	32 (5)	0.06
EDt (ms)	179.95 ± 60	184.72 ± 65	230.35 ± 74	0.05	162 ± 6	171 ± 8	177 ± 7	0.06
IVRT (ms)	119.5 ± 74	120 ± 44	123 ± 48	0.04	57 ± 2	61 ± 4	63 ± 2	0.03
E/A	1.6 ± 0.4	1.54 ± 0.5	1.47 ± 0.7	0.07	2.1 ± 0.3	2.0 ± 0.2	1.8 ± 0.4	0.04
Ea/Aa	1.2 ± 0.2	1.31 ± 0.3	1.38 ± 0.2	0.08	1.1 ± 0.3	1.2 ± 0.3	1.3 ± 0.4	0.07

LV—left ventricle; IVS—interventricular septum thickness; LVEF—left-ventricular ejection fraction; IVRT—isovolumetric relaxation time; Ea—early-diastolic velocity; Aa—late-diastolic velocity; EDt—E-wave deceleration time. ^1^ ANOVA test.

## Data Availability

All data generated or analyzed during the study are included in the published article.

## Abbreviations

AS	Aortic stenosis
AVR	Aortic valve replacement
LV	Left ventricle
LVDFP	Left-ventricular diastolic filling pattern
LA	Left atrium
LVEF	Left-ventricular ejection fraction
LVESD	Left-ventricular end-systolic diameter
NYHA	New york heart association
TDI	Tissue doppler imaging
LVEDV	Left-ventricular end-diastolic volume
LVEDD	Left-ventricular end-diastolic diameter
EDt	E-wave deceleration time
IVRT	Isovolumetric relaxation time
LVOT	Left-ventricular outflow tract
Sa	Peak annular systolic velocity
Ea	Early-diastolic velocity
Aa	Late-diastolic velocity
BNP	Brain natriuretic peptide
ANOVA	Analysis of variance
COPD	Chronic obstructive pulmonary disease
DM	Diabetes mellitus
SR QOL	Self-reported quality of life
IVS	Interventricular septum
ROC	Receiver operating curve
PAP	Pulmonary arterial pressure
